# Effect of Ultrasound‐Guided External Oblique Intercostal Block on Postoperative Recovery After Subxiphoid Video‐Assisted Thoracoscopic Thymectomy: A Randomized Controlled Trial

**DOI:** 10.1002/jum.16591

**Published:** 2024-10-03

**Authors:** Zhiang Li, Lihong Hu, Yong Xi, Lingzhi Wang, Xuwei Zhang, Joseph Mugaanyi

**Affiliations:** ^1^ Department of Anesthesiology The Affiliated Lihuili Hospital of Ningbo University Ningbo China; ^2^ Department of Thoracic Surgery The Affiliated Lihuili Hospital of Ningbo University Ningbo China; ^3^ Department of Hepato‐Pancreato‐Biliary Surgery The Affiliated Lihuili Hospital of Ningbo University Ningbo China; ^4^ Health Science Center Ningbo University Ningbo China

**Keywords:** external oblique, intercostal block, postoperative analgesia, thoracoscopy, thymectomy

## Abstract

**Objective:**

Severe postoperative pain can occur after subxiphoid video‐assisted thoracoscopic thymectomy (SVATT), affecting the quality of postoperative recovery. This study aimed to evaluate the effect of ultrasound‐guided external oblique intercostal (EOI) block on recovery after SVATT.

**Methods:**

A total of 60 patients undergoing SVATT were randomly divided into the EOI group (group E, n = 30) and the control group (group C, n = 30). Group E underwent ultrasound‐guided bilateral EOI block at the 6th rib level and was injected 20 mL of 0.375% ropivacaine on each side. Group C was injected with 20 mL of 0.9% saline at the same site. After the operation, both groups received a patient‐controlled intravenous analgesic (PCIA) pump. The 15‐item Quality of Recovery (QoR‐15) scores were recorded at 24 hours before surgery (T0), 24 hours after surgery (T3), and 48 hours after surgery (T4). The sufentanil usage in the first 24 hours postoperatively, the remifentanil dosage during surgery, the time of first pressing PCIA, and the cases of rescue analgesia were recorded. The visual analog scale (VAS) scores of patients at 6 (T1), 12 (T2), 24 (T3), and 48 hours (T4) after an operation during rest and coughing were recorded. The dermatomes of the sensory plane, block complications in group E, and the incidence of other postoperative adverse reactions in both groups were also recorded.

**Results:**

Compared with group C, the QoR‐15 scores of patients were significantly higher at T3 and T4 in the group E. The VAS scores were significantly lower at T1, T2, and T3 during rest and coughing in the group E. The sufentanil usage in the first 24 hours postoperatively, the remifentanil dosage during surgery, and the cases of rescue analgesia were significantly lower in group E, and the time of first pressing PCIA was significantly increased in group E (all *P* < .05).

**Conclusion:**

Ultrasound‐guided EOI block can be safely used in patients undergoing SVATT, which can improve the quality of postoperative recovery and reduce postoperative pain.

**Abbreviations**
BIS: bispectral index
EOI: external oblique intercostal
PCIA: patient‐controlled intravenous analgesic
QoR‐15: 15‐item Quality of Recovery
SVATT: subxiphoid video‐assisted thoracoscopic thymectomy
TAP: transversus abdominis plane
VAS: visual analog scale

The tumors in the anterior mediastinum are mainly thymomas. In the past, thymectomy was usually performed through median thoracotomy or unilateral thoracoscopic surgery. There were complications such as large surgical trauma, heavy bleeding, arrhythmia, and intercostal nerve damage.^1^ Research has shown that the subxiphoid thoracoscopy surgical method has the advantages of less trauma, a clear surgical field, and a wide resection range, it has now become the mainstream surgical method for thymoma resection.^2^ Regardless of whether there is a single incision under the subxiphoid or multiple incisions under the subxiphoid and costal margin, patients may still suffer from severe postoperative pain due to the surgical operation, which will inhibit active coughing and affect the quality of postoperative recovery.^3^, ^4^ Therefore, the issue of postoperative analgesia in this approach to surgery cannot be ignored. As an alternative to epidural analgesia, ultrasound‐guided fascial plane block avoids the risks of epidural puncture hematoma, hypotension, and local anesthetic poisoning, among others.^5^ The local anesthetic injected into the relevant fascial planes could provide some analgesia in various anatomical areas.^6^, ^7^ Among them, the external oblique intercostal (EOI) block is a novel fascial plane block. Cadaveric and some cases have shown that EOI can block the upper abdominal wall and thoracoabdominal junction area.^8^ Previously, this area could not be completely covered by a single plane block. In addition, EOI also has the advantages of a more convenient bilateral block and an accurate analgesic effect.^9^ We hypothesize that ultrasound‐guided EOI block would improve the quality of postoperative recovery and pain score after subxiphoid video‐assisted thoracoscopic thymectomy (SVATT). This study aims to evaluate the impact of ultrasound‐guided EOI on the postoperative recovery of patients undergoing laparoscopic thymectomy with the subxiphoid approach.

## Materials and Methods

### Patients

**Figure 1:**
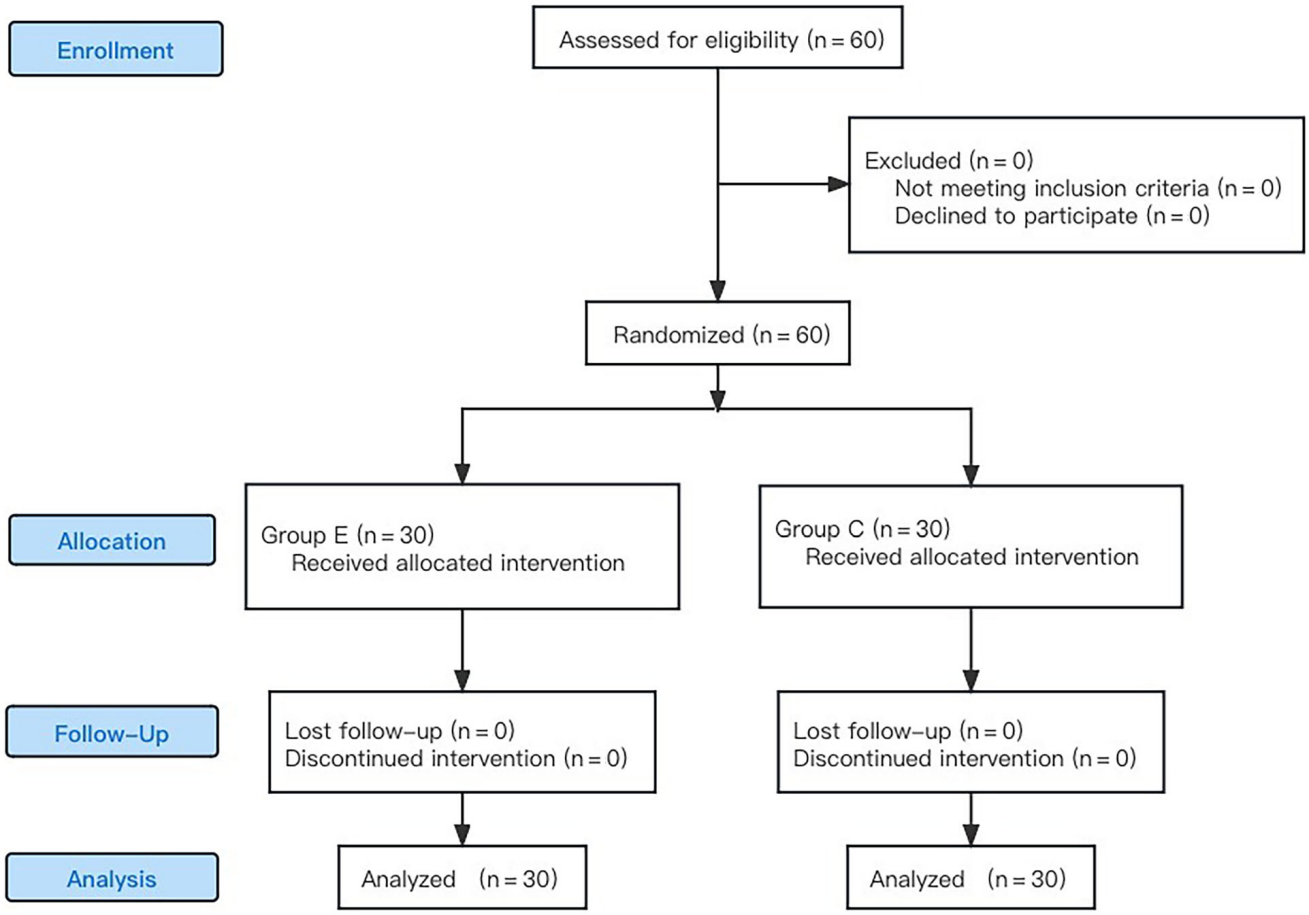
Participant inclusion flow chart.

The hospital's Ethics Committee approved this single‐center, randomized controlled clinical investigation under the following approval code: KY2022SL462‐02. The study was performed in accordance with the Helsinki Declaration of 1964. Ahead of the trial, it was registered on the China Clinical Trial Research website (ChiCTR2300068374), and the subjects signed an informed consent, 60 patients underwent SVATT from January 2023 to September 2023 in Li Huili Hospital of Ningbo Medical Center were enrolled. The patients were randomly allocated into two groups: the EOI group (group E) and the control group (group C) (Figure 1). All nurses and surgeons had the same level of training, and postoperative data were gathered by an anesthesiologist who was uninformed of the patients' categorization. Participants were included based on the following inclusion criteria were as follows: aged between 31 and 69; American Society of Anesthesiologists I–II; and body mass index <28 kg/m^2^. The exclusion criteria included myasthenia gravis syndrome, local anesthetic allergy, severe coagulation disorder, obesity, history of chronic pain, alcoholism, drug abuse, and mental illness. Elimination criteria: those who were converted to thoracotomy during the operation and transferred to the intensive care unit after the operation, and those who were unable to cooperate with the visual analog scale (VAS) score after the operation.

### Analgesia Method

**Figure 2:**
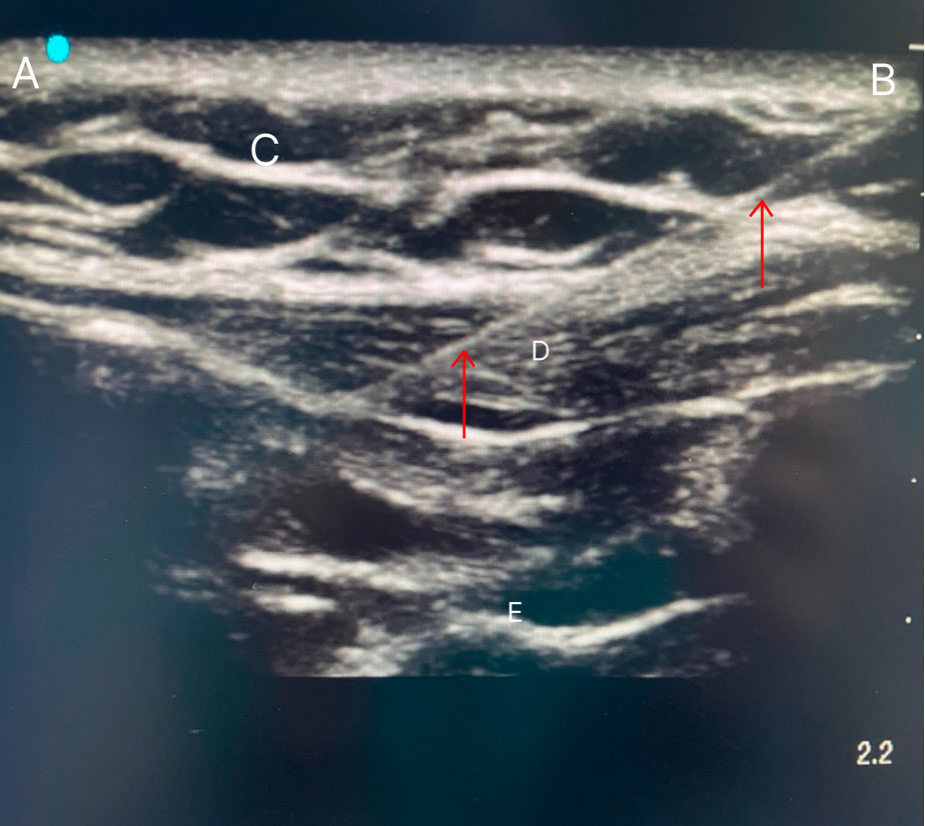
Ultrasonographic image of EOI. **A**, Medial aspect; **B**, lateral aspect; **C**, subcutaneous fat; needle (red arrows); **D**, EO; **E**, pleura.

**Figure 3:**
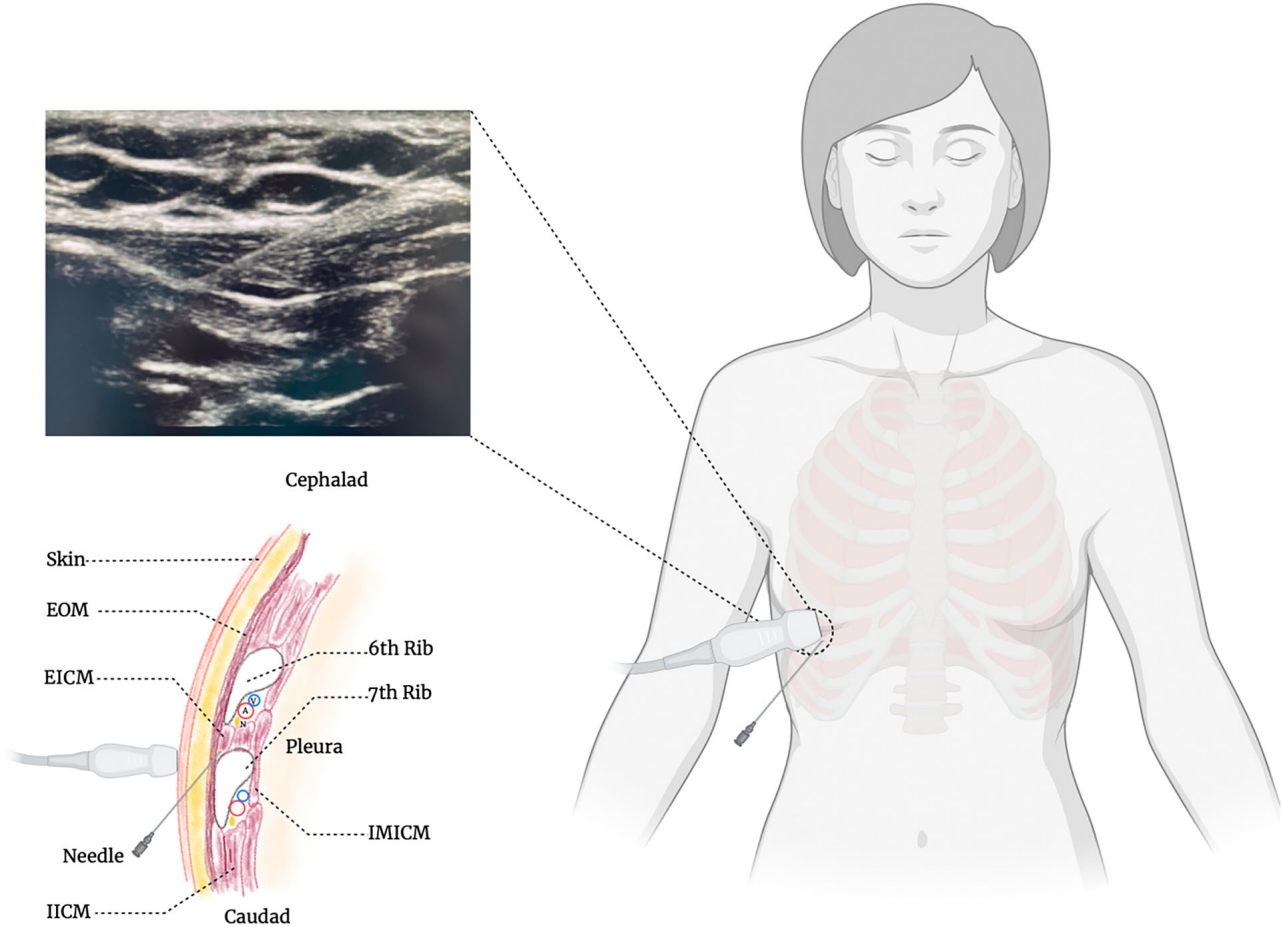
Illustration of the EOI block procedure. EOM, external oblique muscle; EICM, external intercoastal muscle; IICM, internal intercoastal muscle; IMICM, innermost intercoastal muscle; V, vein; A, artery; N, nerve.

After entering the anesthesia preparation room, peripheral venous access was established first then left radial artery and right internal jugular vein access was obtained under local anesthesia. In group E, 2 senior anesthesiologists used ultrasound guidance to place the linear array ultrasound probe (12–15 MHz, SonoSite, USA) between the midclavicular line and the anterior axillary line at the level of the 6th rib, with the direction mark pointing to the cephalad, and the probe was rotated on the cranial end was slightly medial and the caudal end was lateral to produce a mesial sagittal oblique view and a short‐axis view of the ribs. The following structures are identified from superficial to deep layers: subcutaneous tissue, external oblique muscle, and intercostal muscles, pleura, and lung. After determining the thoracic fascial space between the external oblique muscle and the intercostal muscles, 0.375% ropivacaine 20 mL (Naropin, AstraZeneca AB Company, Sweden, 10 mL/75 mg) was slowly injected into the space with a 17‐gauge Tuohy needle at the head side of the 6th rib horizontally near the mid axillary line. The block is repeated in the same way on the other side (Figures 2 and 3). The dosage of ropivacaine adjusted based on individual weight to avoid toxicity. Group C was injected with an equal amount of 0.9% sodium chloride. Anesthesiologists, researchers, and patients were blinded to allocation. The dermatomes of the sensory plane were assessed 15 minutes after block by lightly stroking the skin with a disposable iodophor cotton swab to check for sensation. Block complications such as hematoma or pneumothorax were also monitored by investigators. Investigators were blinded to the group allocation of the participants.

### Anesthesia Protocol and Surgery

Upon entering the operating room, patients were routinely monitored using electrocardiogram, heart rate, percutaneous oxygen saturation (SpO_2_), bispectral index (BIS), and invasive arterial blood pressure. The anesthesia induction plan included intravenous injections of midazolam (0.04 mg/kg), sufentanil (0.5 μg/kg), propofol (2 mg/kg), and rocuronium (0.6 mg/kg). After 2 minutes of controlled breathing with a mask, a video laryngoscope was used to fully expose the glottis. Three milliliters of 2% lidocaine were sprayed for topical anesthesia before intubating the endotracheal tube.

Before the operation, the surgeon verified the patient's position and performed sterile draping. A 3.0‐cm longitudinal incision was made under the xiphoid process (approximately T7 level) to serve as a thoracoscopic observation hole. Additionally, 0.5 cm incisions were made at the costal margin of the bilateral midclavicular line (approximately T8 level) to serve as operating holes. To establish an artificial carbon dioxide pneumothorax, intraoperative air pressure was limited to 8 mmHg.

A total or partial thymectomy was performed. At the end of the operation, 2 chest drainage tubes were placed in the subcostal incision. During anesthesia, volume‐controlled ventilation mode was used with an inspired oxygen concentration of 65%, a tidal volume of 6–8 mL/kg, and positive end expiratory pressure of 0–5 cmH_2_O. The respiratory rate and minute ventilation were adjusted to maintain an end‐tidal carbon dioxide pressure (PET CO_2_) between 30 and 40 mmHg. To maintain an intraoperative BIS value of 40–60, propofol was administered intravenously at 4–12 mg/kg/hour and remifentanil at 0.1–0.2 μg/kg/hour. Rocuronium bromide was given intermittently every 30 minutes to maintain muscle relaxation. If the mean arterial pressure fluctuated by more than 20% from the baseline value, appropriate vasoactive or antihypertensive drugs were administered.

### PCIA and Rescue Analgesia

When the thoracic drainage tube was placed, both groups received 0.15 μg/kg of sufentanil and 3 mg of tropisetron. At the end of the surgery, a patient‐controlled intravenous analgesic (PCIA) pump was connected to the patients. The PCIA pump administered 1.5 μg/kg of sufentanil and 7 mg of tropisetron diluted to 100 mL with normal saline. The background dose was set to 2 mL/hour, with a single extra dose of 2 mL and a lockout time of 15 minutes. The patients were then transferred to the postanesthesia care unit where they were routinely monitored for respiratory antagonism, and the endotracheal tube was withdrawn once the patients were fully conscious and ready for extubation. After safely returning to the ward, if the VAS scores were greater than 3 at rest or greater than 4 when coughing, patients were instructed to press the PCIA self‐control button. If they still felt pain after 5 minutes, 40 mg of parecoxib sodium was administered intravenously for additional analgesia.

### Data Collection

The primary outcome was the 15‐item Quality of Recovery (QoR‐15) scores at 24 hours before operation (T0), 24 hours after operation (T3), and 48 hours after operation (T4). The other outcomes were the VAS scores of patients in the 2 groups at 6 (T1), 12 (T2), 24 (T3), and 48 hours (T4) after operation during rest and coughing, the time of first pressing PCIA, the sufentanil usage in the first 24 hours after operation, the remifentanil dosage during surgery and the cases of rescue analgesia. The dermatomes of the sensory plane and block complications of patients in group E, and the incidence of adverse reactions in the two groups were also recorded. The flowchart in Figure 2 shows the study's participant inclusion.

### Sample Size

The primary outcome of QoR‐15 scores was used to determine the sample size. A difference of at least 8.0 points in the score is considered clinically significant. A total of 10 cases in the two groups participated in the Pilot testing, the QoR‐15 scores at the T3 for the E and C groups were 104.2 (11.8) and 115.3 (12.5), respectively. The sample size was calculated to be 24 patients per group using PASS15.0 with *α* = 0.05, *β* = 0.9. A 20% dropout rate was taken into consideration, and the sample size was increased to 60 patients (30 in each group).

### Statistical Analysis

SPSS version 26.0 was used for the statistical analysis (IBM Corp., Armonk, NY, USA). Normality was tested using the Shapiro–Wilk test. Continuous variables conforming to normal distribution were represented as mean ± standard deviation and were analyzed using the *t* test. Repeated measures data were analyzed using analysis of variance. The skewed distribution data were reported as medians (interquartile range) and were tested using the Kruskal–Wallis *H* test. The Fisher's exact test or the chi‐square test was used for categorical data, which were presented as percentages (%). A *P* < .05 was considered statistically significant.

## Results

This study involved 60 patients in total, with group C serving as the control. Before the operation, all of the patients in the group E had been successfully blocked. Between the two groups, there was no apparent variation in general condition indicators and perioperative characteristics (all *P* > .05; Table 1). The postoperative pathological types included thymoma in 35 cases and thymic cyst in 25 cases.

**Table 1:** General Condition Indicators and Perioperative Characteristics

	Group E (n = 30)	Group C (n = 30)	*P‐*Value
Age (years)	53.9 ± 10.7	53.7 ± 10.6	.933
Gender			
Male	15 (50.0%)	16 (53.3%)	.796
Female	15 (50.0%)	14 (46.7%)	
ASA			
I	19 (63.3%)	20 (66.7%)	.787
II	11 (36.7%)	10 (33.3%)	
BMI (kg/m^2^)	24.0 ± 2.4	23.5 ± 2.7	.430
Type of disease			
Thymoma	18 (60.0%)	17 (56.7%)	.793
Thymic cyst	12 (40.0%)	13 (43.3%)	
Operative time (minutes)	116.8 ± 12.5	114.5 ± 13.3	.482

Data are presented as mean ± standard deviation or as number (%). ASA, American Society of Anesthesiologists; BMI, body mass index.

### Primary Outcome

The QoR‐15 scores were significantly lower at T3 and T4 after surgery than at T0 in both the group E and C. Compared with group C, the QoR‐15 scores of patients were significantly higher at T3 and T4 after surgery in the group E (*P* < .05; Table 2).

**Table 2:** Comparison of QoR‐15 Scores Between Two Groups

	Group E (n = 30)	Group C (n = 30)	Difference (95% CI)	*P‐*Value
T0	124.2 ± 4.7	125.7 ± 5.1	1.5 (−1.0 to 4.0)	.239
T3	108.4 ± 10.2**	99.7 ± 8.4**	−8.6 (−13.5 to −3.8)	.001*
T4	118.1 ± 12.7**	111.6 ± 11.2**	−6.6 (−12.8 to 0.4)	.038*

Data are presented as mean ± standard deviation. Compared with the group C, *P < .05; compared with the T0, **P < .05.

### Other Outcomes

The VAS score during rest and coughing at T4 of the group E and C were significantly lower than that at T1, T2, and T3 after operation (*P* < .05). Compared with the C group, the VAS scores during rest and coughing at T1, T2, and T3 after surgery were significantly lower in group E (*P* < .05; Table 3). There was no significant difference in VAS scores during rest and coughing at T4 after surgery between groups E and C (*P* > .05; Table 3).

**Table 3:** The VAS Score After Surgery

	Group E (n = 30)	Group C (n = 30)	*P*‐Value
VAS at rest			
T1	2.3 ± 0.7	3.2 ± 0.6	<0.001*
T2	2.1 ± 0.6	3.2 ± 0.6	<0.001*
T3	2.0 ± 0.6	3.0 ± 0.6	<0.001*
T4	1.7 ± 0.6	1.9 ± 0.4	0.133
*F*	9.1	99.0	
*P*	<.001*	<.001*	
VAS at coughing			
T1	2.6 ± 0.6	4.1 ± 0.5	<0.001*
T2	2.3 ± 0.5	3.8 ± 0.5	<0.001*
T3	2.2 ± 0.5	3.4 ± 0.6	<0.001*
T4	1.8 ± 0.6	2.0 ± 0.5	0.111
*F*	14.4	125.3	
*P*	<.001*	<.001*	

Data are presented as mean ± standard deviation. Compared with the C group, *P < .001; T4 versus T1, T2, T3, ^Δ^ *P* < 0.001.

The remifentanil dosage during surgery and the sufentanil usage in the first 24 hours postoperatively in group E were significantly lower than those in group C (*P* < .05). The first compression time of the PCIA pump was longer and the cases of rescue analgesia were lower in group E (*P* < .05). There was no significant difference in the incidence of adverse effects among the two groups (*P* > .05; Table 4).

**Table 4:** The Anesthetic Variables and the Adverse Effects

	Group E (n = 30)	Group C (n = 30)	*P*‐Value
Sufentanil usage (μg)	50.1 ± 8.5	55.3 ± 8.8	.022*
Remifentanil dosage (mg)	0.8 ± 0.2	1.1 ± 0.3	<.001*
Time of first pressing PCIA (minutes)	494.1 ± 31.7	417.7 ± 18.8	<.001*
Cases of rescue analgesia	2 (6.7%)	9 (30.0%)	.020*
PONV	5 (16.7%)	8 (26.7%)	.347
Pruritus	4 (13.3%)	6 (20.0%)	.488
Respiratory depression	0	0	

Data are presented as median (interquartile range) or mean ± standard or number (%). Compared with the group C, *P < .05.

**Figure 4:**
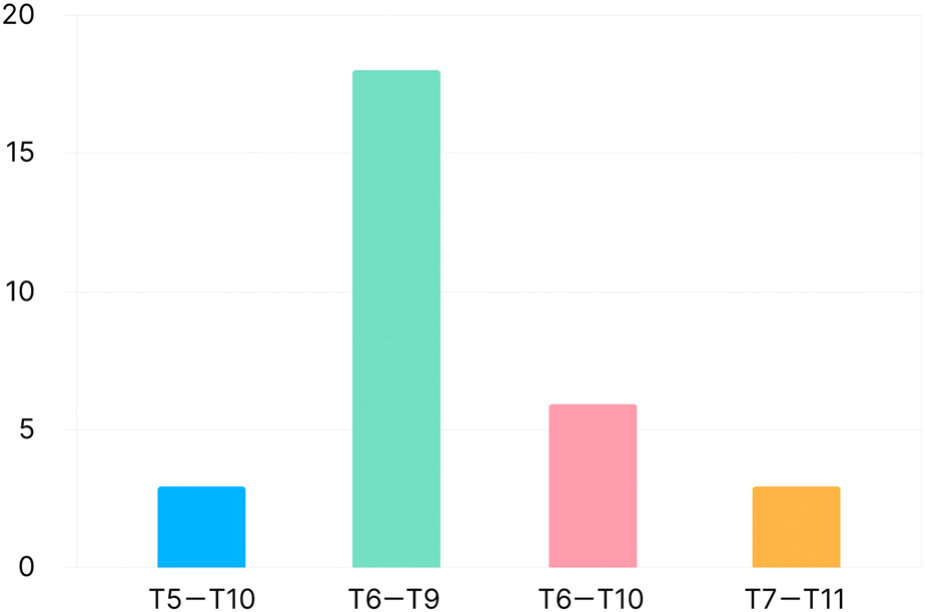
Dermatomal spread in group E.

In group E, the dermatomes of the sensory plane reached T5–T11, the main blocking area was at T6–T9 with 18 cases (60%) (Figure 4), and no block complications such as hematoma or pneumothorax occurred.

## Discussion

When the patient coughs or gets out of bed in the first few days following SVATT, the incision is stressed and the pain is still tremendous.^10^ This trial's purpose is to offer an efficient analgesic for this particular type of operation. Although epidural analgesia continues to be deemed the gold standard for major abdominal and thoracic surgery, it may not always be advantageous for relieving pain because of its association with hypotension and other serious complications, especially in the era of multimodal analgesia and quick recovery procedures.^5^, ^11^ In this study, time *t*_0_ was the preoperative baseline. At *t*_0_, there was no significant difference in QoR‐15 and VAS between the groups implying no difference at the baseline. *t*_1_ − *t*_4_ were the different postoperative observation points. We found significant differences between the groups when compared at these observation points.

Among the known fascial plane blocks, only the subcostal transversus abdominis plane (TAP) block and rectus sheath block can block the area above the umbilicus.^12^, ^13^ The innervation of this area mainly comes from the intercostal nerves from T6 to T10, but according to the shape characteristics of the intercostal nerve and the location of the surgical incision, subcostal TAP is not necessarily suitable for postoperative analgesia during thoracoscopic thymectomy through the xiphoid approach. In addition, a large amount of local anesthetics were required for good diffusion of subcostal TAP block, which increases the risk of local anesthetics poisoning to some extent.

The deep part of the external oblique muscle is continuous with the tissue plane of the deep part of the serratus anterior and latissimus dorsi muscles.^14^, ^15^ Theoretically, a more medial injection around the midclavicular line can block the anterior and lateral branches of the intercostal nerve to provide analgesia for the superior abdominal wall or even chest incisions. Combined with the previous study by Hamilton on a cadaver, the dye was injected into the pectoralis fascial plane deep to the external oblique muscle above the costal margin to stain the lateral and anterior cutaneous branches of the T7–T11 intercostal nerves.^16^ Therefore, the 6th rib of the midclavicular line was finally selected for drug injection in this study, and the puncture site did not affect the subsequent operation but also met the analgesic requirements of the subxiphoid and subcostal incision as far as possible.^17^, ^18^ According to this trial, the dermatomes of the sensory plane of group E were mostly located between T6 and T9, fully covering the subxiphoid incision and drainage tube placement area, and no block‐related complications occurred.

How to effectively evaluate the postoperative recovery of patients has always been the focus of anesthesiologists and surgeons, so we choose the scoring standard of postoperative recovery quality. It is an important indicator of patients' early postoperative health status. Compared with the QoR‐40 score, QoR‐15 is more conducive to preoperative education. The assessment content also includes pain, physical comfort, emotional state, physical independence, and psychological support aspects.^19^, ^20^ In this trial, the QoR‐15 scores at T3 and T4 in group E were significantly higher than those in group C, which proves that a good nerve block effect can promote recovery after surgery. Among them, the difference in QoR‐15 between the two groups at T3 was mainly because EOI block could directly relieve postoperative pain, while the difference in QoR‐15 between the two groups at T4 was still different, which may be the comprehensive result of the choice of blocking drugs, the use of PCIA pump and the action of other drugs, which indirectly improved the postoperative recovery quality of patients to some extent.

In terms of analgesia drug use, compared with group C, the remifentanil dosage during surgery and the sufentanil usage in the first 24 hours postoperatively in group E were significantly lower, possibly because EOI blocked the conduction of pain, thus reducing the surgical stress response, which ultimately reduced the total amount of analgesia drug used. In terms of postoperative analgesia, patients in group E showed lower VAS scores at T1–T3, and incision force in cough state showed better dynamic scores in group E, which further indicated that EOI combined with PCIA had better early postoperative analgesia effect and improved postoperative recovery quality. This fully showed that the postoperative pain of group E was significantly reduced, and once again proved that the ultrasound‐guided EOI block was indeed effective and feasible. In addition, the cases of rescue analgesia in group E were less and the first time of PCIA compression was longer than that in group C, which again demonstrated that the effect of multi‐modal combined analgesia led by nerve block was stronger than that of PCIA pump alone. In terms of the incidence of adverse reactions, the incidence of postoperative nausea and vomiting (PONV) and pruritus in group E was lower than that in group C, but the difference was not statistically significant. The author speculated that the first reason was the small sample size, and the second reason was that EOI could not directly and effectively improve the incidence of PONV and pruritus, but indirectly reduce their occurrence through reducing the use of opioids.

In this trial, when performing the EOI block under ultrasound, the fascial space under the external oblique muscle can be displayed, and the intercostal blood vessels are selectively avoided. The needle entry point was also located at the distal end of the surgical incision. Bilateral EOI block can be completed in the supine position, the patient does not need to repeatedly change positions like serratus anterior plane block, and the comfort level is also high.^18^ In addition, White and other scholars also applied EOI to obese patients and pediatrics, and the effect was very obvious.^21^, ^22^ This shows that EOI has a wide range of applications and is more conducive to clinical promotion and use.

However, this trial still has many shortcomings: 1) This study is a single‐center randomized controlled trial, and the results still need to be verified with large samples from multiple centers. 2) Based on the research of other scholars, the setting criteria for local anesthetics use should first meet surgical requirements, so the optimal use of EOI has not been determined. 3) Studies have shown that using dexamethasone and other drugs around nerves can prolong the action time of nerve block.^23^ This theoretical basis can be an important research direction in the future of this study.

## Conclusion

In conclusion, ultrasound‐guided EOI can improve the quality of postoperative recovery and reduce postoperative pain when applied to patients undergoing SVATT with artificial pneumothorax. The effect was better than that of PCIA alone, without related adverse complications.

## Data Availability

The data that support the findings of this study are available on request from the corresponding author. The data are not publicly available due to privacy or ethical restrictions.
